# Supplementary material for: Spatial Variation in Stream-Water Bacterial Communities Across Sites Differing in Wild Chinese Giant Salamander Detection Frequency
Source: Biology (Basel). 2026 Sep 18;15(18):1647. doi: 10.3390/biology15181647 (PMC13604818; doi:10.3390/biology15181647)
Supplement: Supplementary file 1 [file biology-15-01647-s001.zip › Data Related to “Spatial Variation in Stream-Water Bacterial Communities across Sites Differing in Wild Chinese Giant Salamander Detection Frequency”/Microbial Data/result/02.FeatureAnalysis/denoisingTable_qzv/sample-frequency-detail.html]

q2\_feature\_table : summarize


- Overview
- Interactive Sample Detail
- Feature Detail

### Plot Controls

| Sample ID | Frequency |
| --- | --- |
